# *catena*-Poly[[[tetra­aqua­cadmium(II)]-μ-3,3′-[*p*-phenyl­enebis(oxymethyl­ene)]bis­(1-pyridinioacetate)] dinitrate hemihydrate]

**DOI:** 10.1107/S1600536810036317

**Published:** 2010-09-15

**Authors:** Hong-Lei Lian, Wei-Cheng Pan

**Affiliations:** a College of Chemical Engineering, Zhengzhou University, Zhengzhou, Henan 450001, People’s Republic of China; b College of Chemical Engineering and Foods, Zhongzhou University, Zhengzhou, Henan 450044, People’s Republic of China

## Abstract

In the title polymeric coordination complex, {[Cd(C_22_H_20_N_2_O_6_)(H_2_O)_4_](NO_3_)_2_·0.5H_2_O}_*n*_, obtained from the self-assembly of the flexible double betaine 3,3′-[*p*-phenyl­enebis(oxymethyl­ene)]bis­(1-pyridinioacetate) with cadmium nitrate, both the octa­hedrally coordinated Cd^II^ cation and the substituted betaine ligand lie on inversion centres. The chains constructed through the *trans*-related acetate groups of the ligand are inter-connected *via* O—H⋯O hydrogen bonds involving coordinated aqua ligands, the nitrate anions and the partial-occupancy (0.25) water mol­ecule of solvation, forming a three-dimensional structure.

## Related literature

For betaine–metal coordination compexes, see: Zhang *et al.* (2004 ▶); Zhang & Mak (2004 ▶). For the structure of the copper(II) complex with the ligand employed here, see: Pan & Lian (2010 ▶).
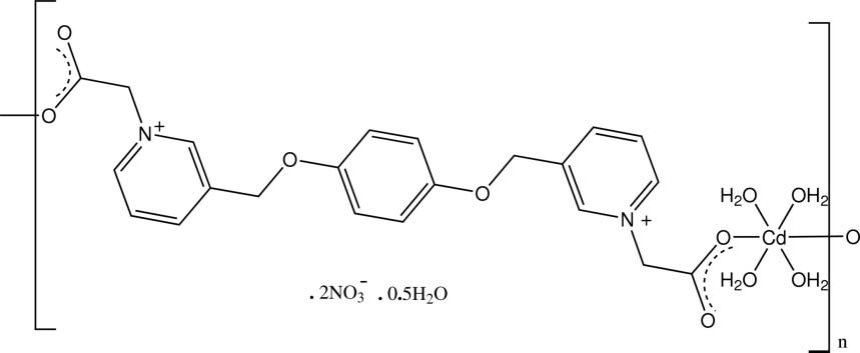


## Experimental

### 

#### Crystal data

[Cd(C_22_H_20_N_2_O_6_)(H_2_O)_4_](NO_3_)_2_·0.5H_2_O
*M* *_r_* = 725.90
Monoclinic, 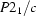

*a* = 6.5627 (17) Å
*b* = 14.612 (2) Å
*c* = 14.9730 (15) Å
β = 91.922 (19)°
*V* = 1435.0 (5) Å^3^
*Z* = 2
Mo *K*α radiation
μ = 0.85 mm^−1^
*T* = 153 K
0.48 × 0.36 × 0.32 mm

#### Data collection

Bruker SMART CCD area-detector diffractometer
Absorption correction: multi-scan (*SADABS*; Sheldrick, 2004 ▶) *T*_min_ = 0.687, *T*_max_ = 0.774
3478 measured reflections
2515 independent reflections
1654 reflections with *I* > 2σ(*I*)
*R*_int_ = 0.037

#### Refinement

*R*[*F* ^2^ > 2σ(*F* ^2^)] = 0.045
*wR*(*F* ^2^) = 0.081
*S* = 1.02
2515 reflections
205 parameters
6 restraints
H-atom parameters constrained
Δρ_max_ = 0.34 e Å^−3^
Δρ_min_ = −0.33 e Å^−3^

### 

Data collection: *SMART* (Bruker, 2001 ▶); cell refinement: *SAINT* (Bruker, 2001 ▶); data reduction: *SAINT*; program(s) used to solve structure: *SHELXS97* (Sheldrick, 2008 ▶); program(s) used to refine structure: *SHELXL97* (Sheldrick, 2008 ▶); molecular graphics: *SHELXTL* (Sheldrick, 2008 ▶); software used to prepare material for publication: *SHELXTL*.

## Supplementary Material

Crystal structure: contains datablocks I, global. DOI: 10.1107/S1600536810036317/zs2059sup1.cif

Structure factors: contains datablocks I. DOI: 10.1107/S1600536810036317/zs2059Isup2.hkl

Additional supplementary materials:  crystallographic information; 3D view; checkCIF report

## Figures and Tables

**Table 1:** Hydrogen-bond geometry (Å, °)

*D*—H⋯*A*	*D*—H	H⋯*A*	*D*⋯*A*	*D*—H⋯*A*
O1*W*—H1*WA*⋯O6	0.85	2.11	2.950 (7)	172
O1*W*—H1*WB*⋯O2^i^	0.85	2.07	2.832 (5)	150
O1*W*—H1*WB*⋯O3*W*^ii^	0.85	2.28	2.835 (16)	123
O2*W*—H2*WA*⋯O2^iii^	0.85	1.93	2.766 (5)	169
O2*W*—H2*WB*⋯O5^iv^	0.85	2.21	3.041 (7)	166
O2*W*—H2*WB*⋯O4^iv^	0.85	2.43	3.122 (6)	139
O3*W*—H3*WA*⋯O2^v^	0.85	2.13	2.886 (14)	149
O3*W*—H3*WA*⋯O2*W*^vi^	0.85	2.55	3.211 (15)	136
O3*W*—H3*WB*⋯O6^vi^	0.85	2.15	2.859 (16)	141

Symmetry codes: (i) 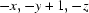; (ii) 
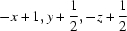
; (iii) 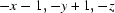; (iv) 
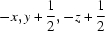
; (v) 
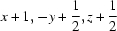
; (vi) 
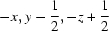
.

**Fig. 1.**
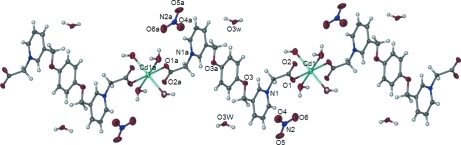
A portion of the infinite chain of the title compound viewed along the a cell direction, showing atom numbering scheme. Atoms are drawn with 30% probability displacement ellipsoids. For symmetry code a: -x + 1, -y, -z.

**Fig. 2.**
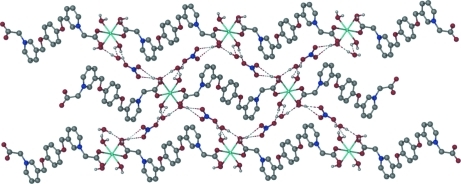
The three-dimensional structure of (I) formed through intermolecular hydrogen bonds, shown as dashed lines.

## supplementary crystallographic information

### Comment

The coordinative bond approach has been widely used in the construction of supramolecular coordination compounds. As a parallel development, it is possible to use highly directional hydrogen bonds as a means of controlling self-assembly. Double betaines, comprising two betaine moieties and having two terminal anionic carboxylate substituent groups are of great utility for linkage to a broad array of metal ions, generating a variey of supramolecular entities, ranging from discrete complex units, through networks linked by hydrogen bonding, to metallo-supramolecular systems (Zhang *et al.*, 2004); Zhang & Mak, 2004). The double betaine ligand 1,4-bis(3-picolyloxyl)benzene-*N*,*N*^'^-diacetate (*L*) has provided the structure of a polymeric complex with Cu^II^ (Pan & Lian, 2010). Our reaction of *L* with cadmium nitrate gave the polymeric coordination complex [[Cd_2_(*L*)(H_2_O)_4_]_n_ 2n(NO_3_) 0.5n(H_2_O)], the title compound (I) and the structure is reported here. In (I), the Cd^II^ cations have octahedral stereochemistry and lie on crystallographic inversion centres and are coordinated by two *trans*-related acetato-O donors [Cd–O, 2.219 (3) Å] and four water molecules [Cd–O, 2.321 (3), 2.324 (4) Å] (Fig. 1). The substituted betaine ligand also lies across a crysallographic inversion centre, forming an infinite zigzag chain structure. These chains are further inter-connected by intermolecular hydrogen-bonding interactions involving the nitrate anions, the coordinated aqua ligands and the partial-occupancy (S.O.F = 0.25) water molecule of solvation (Table 1), to form a three-dimensional structure (Fig. 2).

### Experimental

An aqueous solution of 1,4-bis(3-picolyloxyl)benzene-*N*,*N*^'^-diacetate (dehydrated) (5 ml; 0.08 g, 0.2 mmol) and cadmium nitrate (0.093 g, 0.3 mmol) were combined and heated at 70° for 30 minutes and then filtered. Colorless block-shaped crystals were obtained upon slow evaporation of the filtrate at room temperature for several weeks (yield: *ca.* 46% based on *L*).

### Refinement

The H atoms of the water molecule were located in a difference map but were constrained in the refinement. Other H atoms were positioned geometrically and refined using a riding model with C—H = 0.93, 0.97 Å and O–H = 0.85 Å, with *U*_iso_(H) = 1.2*U*_eq_(C).

### Crystal data

**Table:**

[Cd(C_22_H_20_N_2_O_6_)(H_2_O)_4_](NO_3_)_2_·0.5H_2_O	*F*(000) = 738
*M**_r_* = 725.90	*D*_x_ = 1.680 Mg m^−^^3^
Monoclinic, *P*2_1_/*c*	Mo *K*α radiation, λ = 0.71073 Å
Hall symbol: -P 2ybc	Cell parameters from 245 reflections
*a* = 6.5627 (17) Å	θ = 2.0–27.5°
*b* = 14.612 (2) Å	µ = 0.85 mm^−^^1^
*c* = 14.9730 (15) Å	*T* = 153 K
β = 91.922 (19)°	Block, colorless
*V* = 1435.0 (5) Å^3^	0.48 × 0.36 × 0.32 mm
*Z* = 2	

### Data collection

**Table:**

Bruker SMART CCD area-detector diffractometer	2515 independent reflections
Radiation source: fine-focus sealed tube	1654 reflections with *I* > 2σ(*I*)
graphite	*R*_int_ = 0.037
φ and ω scans	θ_max_ = 25.0°, θ_min_ = 2.0°
Absorption correction: multi-scan (*SADABS*; Sheldrick, 2004)	*h* = −1→7
*T*_min_ = 0.687, *T*_max_ = 0.774	*k* = −1→17
3478 measured reflections	*l* = −17→17

### Refinement

**Table:**

Refinement on *F*^2^	Primary atom site location: structure-invariant direct methods
Least-squares matrix: full	Secondary atom site location: difference Fourier map
*R*[*F*^2^ > 2σ(*F*^2^)] = 0.045	Hydrogen site location: inferred from neighbouring sites
*wR*(*F*^2^) = 0.081	H-atom parameters constrained
*S* = 1.02	*w* = 1/[σ^2^(*F*_o_^2^) + (0.0152*P*)^2^ + 1.4665*P*] where *P* = (*F*_o_^2^ + 2*F*_c_^2^)/3
2515 reflections	(Δ/σ)_max_ < 0.001
205 parameters	Δρ_max_ = 0.34 e Å^−^^3^
6 restraints	Δρ_min_ = −0.33 e Å^−^^3^

### Special details

**Table:**

Geometry. All e.s.d.'s (except the e.s.d. in the dihedral angle between two l.s. planes) are estimated using the full covariance matrix. The cell e.s.d.'s are taken into account individually in the estimation of e.s.d.'s in distances, angles and torsion angles; correlations between e.s.d.'s in cell parameters are only used when they are defined by crystal symmetry. An approximate (isotropic) treatment of cell e.s.d.'s is used for estimating e.s.d.'s involving l.s. planes.
Refinement. Refinement of *F*^2^ against ALL reflections. The weighted *R*-factor *wR* and goodness of fit *S* are based on *F*^2^, conventional *R*-factors *R* are based on *F*, with *F* set to zero for negative *F*^2^. The threshold expression of *F*^2^ > σ(*F*^2^) is used only for calculating *R*-factors(gt) *etc*. and is not relevant to the choice of reflections for refinement. *R*-factors based on *F*^2^ are statistically about twice as large as those based on *F*, and *R*- factors based on ALL data will be even larger.

### Fractional atomic coordinates and isotropic or equivalent isotropic displacement parameters (Å^2^)

**Table:**

	*x*	*y*	*z*	*U*_iso_*/*U*_eq_	Occ. (<1)
Cd1	0.0000	0.5000	0.0000	0.04421 (19)	
N2	0.0260 (8)	0.3049 (4)	0.3014 (3)	0.0512 (13)	
O4	0.1818 (7)	0.2675 (4)	0.2799 (3)	0.0978 (17)	
O5	−0.0681 (8)	0.2698 (4)	0.3614 (3)	0.111 (2)	
O6	−0.0265 (9)	0.3747 (4)	0.2635 (4)	0.113 (2)	
N1	−0.3871 (6)	0.2278 (3)	0.0648 (2)	0.0288 (9)	
O1	−0.2220 (5)	0.3883 (2)	0.0220 (2)	0.0481 (10)	
O2	−0.4430 (5)	0.4167 (2)	−0.0904 (2)	0.0446 (9)	
O3	0.1499 (5)	0.0941 (2)	0.0419 (2)	0.0441 (9)	
O1W	0.2047 (5)	0.4226 (2)	0.1045 (3)	0.0592 (11)	
H1WA	0.1390	0.4146	0.1518	0.071*	
H1WB	0.2900	0.4640	0.1198	0.071*	
O2W	−0.1374 (5)	0.5844 (2)	0.1150 (2)	0.0579 (11)	
H2WA	−0.2639	0.5886	0.1015	0.069*	
H2WB	−0.1002	0.6395	0.1239	0.069*	
C1	−0.3701 (7)	0.3696 (3)	−0.0283 (3)	0.0322 (12)	
C2	−0.4697 (7)	0.2772 (3)	−0.0141 (3)	0.0349 (13)	
H2A	−0.6149	0.2862	−0.0077	0.042*	
H2B	−0.4516	0.2397	−0.0668	0.042*	
C3	−0.2120 (7)	0.1805 (3)	0.0579 (3)	0.0311 (11)	
H3A	−0.1448	0.1803	0.0042	0.037*	
C4	−0.1326 (7)	0.1328 (3)	0.1296 (3)	0.0284 (11)	
C5	−0.2363 (8)	0.1349 (3)	0.2090 (3)	0.0382 (12)	
H5A	−0.1861	0.1026	0.2585	0.046*	
C6	−0.4124 (8)	0.1845 (4)	0.2142 (3)	0.0424 (14)	
H6A	−0.4818	0.1864	0.2673	0.051*	
C7	−0.4854 (7)	0.2311 (3)	0.1412 (3)	0.0366 (12)	
H7A	−0.6043	0.2654	0.1447	0.044*	
C8	0.0605 (7)	0.0779 (4)	0.1255 (3)	0.0411 (13)	
H8A	0.1545	0.0958	0.1737	0.049*	
H8B	0.0305	0.0133	0.1319	0.049*	
C9	0.3246 (7)	0.0448 (3)	0.0241 (3)	0.0321 (12)	
C10	0.4000 (7)	0.0579 (3)	−0.0602 (3)	0.0350 (12)	
H10A	0.3327	0.0966	−0.1006	0.042*	
C11	0.4228 (7)	−0.0127 (4)	0.0841 (3)	0.0364 (12)	
H11A	0.3712	−0.0215	0.1406	0.044*	
O3W	0.424 (2)	−0.0647 (10)	0.2940 (8)	0.059 (4)	0.25
H3WA	0.4185	−0.0133	0.3204	0.070*	0.25
H3WB	0.3284	−0.1040	0.2933	0.070*	0.25

### Atomic displacement parameters (Å^2^)

**Table:**

	*U*^11^	*U*^22^	*U*^33^	*U*^12^	*U*^13^	*U*^23^
Cd1	0.0345 (3)	0.0305 (3)	0.0675 (4)	−0.0037 (4)	0.0010 (3)	0.0071 (4)
N2	0.053 (3)	0.055 (3)	0.046 (3)	−0.005 (3)	0.003 (3)	−0.017 (3)
O4	0.076 (3)	0.102 (4)	0.116 (4)	0.035 (3)	0.028 (3)	−0.002 (3)
O5	0.129 (5)	0.139 (5)	0.070 (3)	−0.062 (4)	0.055 (3)	−0.036 (3)
O6	0.153 (5)	0.070 (3)	0.114 (4)	0.043 (4)	−0.018 (4)	0.003 (3)
N1	0.026 (2)	0.030 (2)	0.031 (2)	−0.0062 (19)	0.0017 (19)	−0.0014 (18)
O1	0.044 (2)	0.045 (2)	0.054 (2)	−0.0203 (19)	−0.011 (2)	0.0134 (19)
O2	0.047 (2)	0.039 (2)	0.048 (2)	−0.0017 (19)	−0.0003 (19)	0.0126 (19)
O3	0.035 (2)	0.049 (2)	0.049 (2)	0.0190 (18)	0.0109 (17)	0.0077 (19)
O1W	0.053 (2)	0.049 (2)	0.075 (3)	0.006 (2)	−0.014 (2)	0.000 (2)
O2W	0.046 (2)	0.052 (2)	0.075 (3)	0.000 (2)	−0.007 (2)	−0.010 (2)
C1	0.028 (3)	0.036 (3)	0.033 (3)	−0.002 (2)	0.006 (2)	−0.003 (2)
C2	0.031 (3)	0.034 (2)	0.040 (3)	−0.001 (3)	−0.003 (3)	0.005 (3)
C3	0.026 (3)	0.032 (3)	0.035 (3)	−0.003 (2)	0.005 (2)	−0.004 (2)
C4	0.024 (2)	0.026 (2)	0.036 (3)	−0.006 (2)	0.003 (2)	−0.004 (2)
C5	0.039 (3)	0.039 (3)	0.036 (3)	0.000 (3)	−0.003 (3)	0.003 (3)
C6	0.045 (3)	0.053 (3)	0.030 (3)	0.000 (3)	0.009 (3)	−0.002 (3)
C7	0.032 (3)	0.037 (3)	0.042 (3)	0.002 (2)	0.013 (3)	−0.005 (3)
C8	0.032 (3)	0.044 (3)	0.048 (3)	0.003 (3)	0.007 (3)	0.005 (3)
C9	0.023 (3)	0.029 (2)	0.045 (3)	0.005 (2)	−0.001 (2)	−0.007 (2)
C10	0.036 (3)	0.029 (3)	0.040 (3)	0.000 (2)	−0.006 (3)	0.000 (2)
C11	0.031 (2)	0.039 (3)	0.039 (3)	0.001 (3)	0.004 (2)	−0.001 (3)
O3W	0.089 (12)	0.050 (9)	0.037 (8)	−0.006 (9)	0.000 (9)	−0.016 (8)

### Geometric parameters (Å, °)

**Table:**

Cd1—O1^i^	2.219 (3)	C2—H2A	0.9700
Cd1—O1	2.219 (3)	C2—H2B	0.9700
Cd1—O1W	2.321 (3)	C3—C4	1.369 (6)
Cd1—O1W^i^	2.321 (3)	C3—H3A	0.9300
Cd1—O2W^i^	2.324 (4)	C4—C5	1.390 (6)
Cd1—O2W	2.324 (4)	C4—C8	1.503 (6)
N2—O6	1.212 (6)	C5—C6	1.369 (6)
N2—O4	1.213 (6)	C5—H5A	0.9300
N2—O5	1.220 (6)	C6—C7	1.360 (7)
N1—C7	1.334 (5)	C6—H6A	0.9300
N1—C3	1.348 (5)	C7—H7A	0.9300
N1—C2	1.472 (6)	C8—H8A	0.9700
O1—C1	1.240 (5)	C8—H8B	0.9700
O2—C1	1.240 (5)	C9—C11	1.375 (6)
O3—C9	1.387 (5)	C9—C10	1.384 (6)
O3—C8	1.420 (5)	C10—C11^ii^	1.394 (6)
O1W—H1WA	0.8501	C10—H10A	0.9300
O1W—H1WB	0.8501	C11—C10^ii^	1.394 (6)
O2W—H2WA	0.8500	C11—H11A	0.9300
O2W—H2WB	0.8500	O3W—H3WA	0.8499
C1—C2	1.518 (6)	O3W—H3WB	0.8500
			
O1^i^—Cd1—O1	180.00 (18)	N1—C2—H2B	108.9
O1^i^—Cd1—O1W	95.17 (13)	C1—C2—H2B	108.9
O1—Cd1—O1W	84.83 (13)	H2A—C2—H2B	107.7
O1^i^—Cd1—O1W^i^	84.83 (13)	N1—C3—C4	120.3 (4)
O1—Cd1—O1W^i^	95.17 (13)	N1—C3—H3A	119.9
O1W—Cd1—O1W^i^	180.0	C4—C3—H3A	119.9
O1^i^—Cd1—O2W^i^	90.43 (13)	C3—C4—C5	118.4 (4)
O1—Cd1—O2W^i^	89.57 (13)	C3—C4—C8	122.6 (4)
O1W—Cd1—O2W^i^	90.61 (13)	C5—C4—C8	119.0 (4)
O1W^i^—Cd1—O2W^i^	89.39 (13)	C6—C5—C4	119.9 (5)
O1^i^—Cd1—O2W	89.57 (13)	C6—C5—H5A	120.1
O1—Cd1—O2W	90.43 (13)	C4—C5—H5A	120.1
O1W—Cd1—O2W	89.39 (13)	C7—C6—C5	119.7 (5)
O1W^i^—Cd1—O2W	90.61 (13)	C7—C6—H6A	120.2
O2W^i^—Cd1—O2W	180.00 (15)	C5—C6—H6A	120.2
O6—N2—O4	119.0 (6)	N1—C7—C6	120.3 (5)
O6—N2—O5	123.8 (6)	N1—C7—H7A	119.9
O4—N2—O5	117.2 (6)	C6—C7—H7A	119.9
C7—N1—C3	121.5 (4)	O3—C8—C4	108.7 (4)
C7—N1—C2	119.7 (4)	O3—C8—H8A	109.9
C3—N1—C2	118.8 (4)	C4—C8—H8A	109.9
C1—O1—Cd1	125.2 (3)	O3—C8—H8B	109.9
C9—O3—C8	116.8 (4)	C4—C8—H8B	109.9
Cd1—O1W—H1WA	109.3	H8A—C8—H8B	108.3
Cd1—O1W—H1WB	101.3	C11—C9—C10	120.4 (4)
H1WA—O1W—H1WB	102.8	C11—C9—O3	124.4 (4)
Cd1—O2W—H2WA	105.3	C10—C9—O3	115.2 (4)
Cd1—O2W—H2WB	120.1	C9—C10—C11^ii^	119.7 (4)
H2WA—O2W—H2WB	103.9	C9—C10—H10A	120.1
O1—C1—O2	127.4 (5)	C11^ii^—C10—H10A	120.1
O1—C1—C2	116.3 (5)	C9—C11—C10^ii^	119.9 (4)
O2—C1—C2	116.2 (4)	C9—C11—H11A	120.1
N1—C2—C1	113.5 (4)	C10^ii^—C11—H11A	120.1
N1—C2—H2A	108.9	H3WA—O3W—H3WB	124.1
C1—C2—H2A	108.9		
			
O1W—Cd1—O1—C1	−163.1 (4)	C8—C4—C5—C6	179.9 (5)
O1W^i^—Cd1—O1—C1	16.9 (4)	C4—C5—C6—C7	0.4 (8)
O2W^i^—Cd1—O1—C1	−72.4 (4)	C3—N1—C7—C6	−1.5 (7)
O2W—Cd1—O1—C1	107.6 (4)	C2—N1—C7—C6	178.9 (4)
Cd1—O1—C1—O2	−13.1 (7)	C5—C6—C7—N1	0.6 (8)
Cd1—O1—C1—C2	164.9 (3)	C9—O3—C8—C4	−176.5 (4)
C7—N1—C2—C1	98.2 (5)	C3—C4—C8—O3	6.8 (6)
C3—N1—C2—C1	−81.5 (5)	C5—C4—C8—O3	−173.7 (4)
O1—C1—C2—N1	6.3 (6)	C8—O3—C9—C11	−4.8 (7)
O2—C1—C2—N1	−175.4 (4)	C8—O3—C9—C10	176.0 (4)
C7—N1—C3—C4	1.3 (7)	C11—C9—C10—C11^ii^	−0.3 (8)
C2—N1—C3—C4	−179.0 (4)	O3—C9—C10—C11^ii^	178.9 (4)
N1—C3—C4—C5	−0.3 (7)	C10—C9—C11—C10^ii^	0.3 (8)
N1—C3—C4—C8	179.2 (4)	O3—C9—C11—C10^ii^	−178.8 (4)
C3—C4—C5—C6	−0.6 (7)		

Symmetry codes: (i) −*x*, −*y*+1, −*z*; (ii) −*x*+1, −*y*, −*z*.

### Hydrogen-bond geometry (Å, °)

**Table:**

*D*—H···*A*	*D*—H	H···*A*	*D*···*A*	*D*—H···*A*
O1W—H1WA···O6	0.85	2.11	2.950 (7)	172
O1W—H1WB···O2^i^	0.85	2.07	2.832 (5)	150
O1W—H1WB···O3W^iii^	0.85	2.28	2.835 (16)	123
O2W—H2WA···O2^iv^	0.85	1.93	2.766 (5)	169
O2W—H2WB···O5^v^	0.85	2.21	3.041 (7)	166
O2W—H2WB···O4^v^	0.85	2.43	3.122 (6)	139
O3W—H3WA···O2^vi^	0.85	2.13	2.886 (14)	149
O3W—H3WA···O2W^vii^	0.85	2.55	3.211 (15)	136
O3W—H3WB···O6^vii^	0.85	2.15	2.859 (16)	141

Symmetry codes:  (i) −*x*, −*y*+1, −*z*; (iii) −*x*+1, *y*+1/2, −*z*+1/2; (iv) −*x*−1, −*y*+1, −*z*; (v) −*x*, *y*+1/2, −*z*+1/2; (vi) *x*+1, −*y*+1/2, *z*+1/2; (vii) −*x*, *y*−1/2, −*z*+1/2.
